# Regret Expression and Social Learning Increases Delay to Sexual Gratification

**DOI:** 10.1371/journal.pone.0135977

**Published:** 2015-08-17

**Authors:** Amanda J. Quisenberry, Celia R. Eddy, David L. Patterson, Christopher T. Franck, Warren K. Bickel

**Affiliations:** 1 Virginia Tech Carilion Research Institute, Roanoke, Virginia, United States of America; 2 Virginia Tech, Department of Statistics, Blacksburg, Virginia, United States of America; Institutes for Behavior Resources and Johns Hopkins University School of Medicine, UNITED STATES

## Abstract

**Objective:**

Modification and prevention of risky sexual behavior is important to individuals’ health and public health policy. This study employed a novel sexual discounting task to elucidate the effects of social learning and regret expression on delay to sexual gratification in a behavioral task.

**Methods:**

Amazon Mechanical Turk Workers were assigned to hear one of three scenarios about a friend who engages in similar sexual behavior. The scenarios included a positive health consequence, a negative health consequence or a negative health consequence with the expression of regret. After reading one scenario, participants were asked to select from 60 images, those with whom they would have casual sex. Of the selected images, participants chose one image each for the person they most and least want to have sex with and person most and least likely to have a sexually transmitted infection. They then answered questions about engaging in unprotected sex now or waiting some delay for condom-protected sex in each partner condition.

**Results:**

Results indicate that the negative health outcome scenario with regret expression resulted in delayed sexual gratification in the most attractive and least STI partner conditions, whereas in the least attractive and most STI partner conditions the negative health outcome with and without regret resulted in delayed sexual gratification.

**Conclusions:**

Results suggest that the sexual discounting task is a relevant laboratory measure and the framing of information to include regret expression may be relevant for prevention of risky sexual behavior.

## Introduction

Modification and prevention of risky sexual behavior is important to both individuals’ health and public health policy. Reducing high risk sexual behavior is one way to prevent the spread of HIV [1]. Recent laboratory studies have shown that responses on a behavioral task measuring risky sexual behavior demonstrate a similar quantitative signature to responses in monetary delay discounting [2], a measure of future valuation [3,4]. A variety of manipulations have changed hypothetical monetary delay discounting [5], which suggests that procedures with a similar quantitative pattern can be changed by related strategies. An important question is whether modifying this extension of delay discounting is viable and will ultimately lead to changes in future sexual behavior.

This procedure for measuring risky sexual behavior, termed sexual discounting, is a valid and reliable way to characterize waiting for condom-protected sex [2,6]. During the sexual discounting task, participants are asked if they would rather have unprotected sex now or wait a certain delay (e.g., an hour, a day, a week) to engage in condom-protected sex. Investigations using this procedure have determined that when compared with control participants, drug-dependent individuals are more likely to choose immediate unprotected sex than wait for delayed condom-protected sex. For example, cocaine-dependent and recreational cocaine users [7], opioid-dependent women [8], and alcohol-dependent participants [9] discount delayed condom-protected sex at higher rates than controls. These observations are consistent with a broad range of studies examining hypothetical monetary discounting between control participants and cocaine- [10,11], opioid- [12,13], and alcohol-dependent participants [14].

Delay discounting of hypothetical monetary rewards can be briefly manipulated by many categories of interventions (for review see [5]). For example, decreased delay discounting rates have been shown after working memory training [15], contingency management [16], transcranial magnetic stimulation [17], and both administration of [18,19] and withdrawal from drugs [20,21]. Framing, or the information presented immediately preceding a decision, has also been shown to change delay discounting. For example, the presence of prospective thought cues [22], explicit framing of a question [23], and prior task completion [24] all alter the subjective value of the presented choices. Moreover, presenting framing information not related to timing before task completion has also been found to change delay discounting. Being presented with information about imaginary financial situations [25] and fair versus unfair circumstances [26] results in changed monetary discounting.

Given the ability to modify monetary delay discounting rate and that discounting for different commodities exhibit hyperbolic shaped functions [27], changing hypothetical sexual discounting by similar antecedent manipulations may be possible. Determining the factors that are able to alter risky sexual behavior is necessary and important for public health efforts to reduce high risk sexual behavior that may contribute to the spread of HIV [1]. Changing hypothetical sexual discounting using antecedent manipulations could test real world interventions that may change risky decision making. Perhaps, this could be accomplished by structuring antecedent statements with positive or negative social learning outcomes. Further, the addition of a decision making bias may potentiate the effect.

Humans are social animals and can learn from direct experience or the actions of conspecifics. For example, in a novel social influence paradigm of delay discounting for monetary rewards, choice between smaller, sooner rewards and larger, delayed rewards was influenced by viewing a peer’s choices prior to answering [28]. Adolescents who viewed peer choices preferring smaller, sooner rewards were more likely to choose smaller, sooner rewards, while those who viewed peer choices preferring larger, delayed rewards were more likely to choose that option. Creating a scenario regarding another may be a way to incorporate social learning into antecedent manipulations.

Among the biases that affect human decision making is regret aversion [29–33], an emotion experienced when imagining that current circumstances would be better if a different decision had been made in the past [34]. Regret theory states that the value of a decision is dependent on the alternatives that are simultaneously rejected [33]. People are likely to make choices in a way that minimize this future regret [34], thus anticipation of regret facilitates decision making with longer forethought [33]. Making individuals aware of future regret has been shown to change purchasing behavior [35], alter attitudes about unsafe driving [36], and result in less self-reported risky sexual behavior post-anticipation [37]. These results suggest that the anticipation of regret results in risk aversion where an individual faced with two choices is more likely to choose the option with a more certain payoff than an uncertain payoff [38]. In sexual decision making, a regret averse individual may be more likely to choose condom-protected sex.

The aim of the current study was to identify how reading scenarios that employ social learning outcomes and a decision making bias would engender choice of less risky sex as measured by the sexual discounting task. We hypothesized that reading a scenario about a similar individual who experienced a negative health consequence of unprotected sexual intercourse will engender greater waiting for condom-protected sex compared to a similar scenario that does not result in a negative health outcome. Moreover, we hypothesized that adding expression of regret to the scenario with the negative health consequence would increase waiting for condom-protected sex compared to the negative health outcome alone.

To test the selectivity of the scenarios on risky sexual behavior, we employed the standard partner conditions of the sexual discounting task; that is, decisions about waiting for condom use occur under four partner conditions consisting of least attractive, most attractive, least likely to have a sexually transmitted infection and most likely to have a sexually transmitted infection. We hypothesized that scenarios with negative health outcomes and regret expression will engender the greatest delay of sexual gratification in the most attractive and least likely to have a sexually transmitted infection partner conditions; that is, under the conditions where participants may be least likely to delay sexual gratification, as demonstrated in two previous studies [2,8].

To examine the relationship between the novel sexual discounting procedure and validated measures of risky sexual behavior, participants also completed questionnaires of risky sexual decision making. Consistent with previous research, we hypothesized that participants with high sexual discounting rates would also show high scores on the HIV Risk Taking Behavior Scale. Finally, selectivity of the scenarios was further tested by examining their effect on the discounting of monetary outcomes. Given the scenarios have no information pertaining to financial situations, we hypothesized that monetary discounting would not be different across the scenarios.

## Materials and Methods

### Participants

Participants were 408 workers from Amazon Mechanical Turk, a crowdsourcing website, who accepted a Human Intelligence Task (HIT) about decision making posted by the Addiction Recovery Research Center. To be included in this study, participants must have been at least 18 years old, resided within the U.S., and had a previous HIT approval rate of 90% or greater. Participants were excluded if they had previously participated in a Virginia Tech funded study. Participants were compensated $1.00 for completion of the study and received a $2.00 bonus if they provided consistent hypothetical monetary delay discounting responses [39].

Participants were randomly assigned to one of three scenario conditions: positive (n = 136), negative (n = 137), and negative with regret expression (n = 135). Statistical analysis on demographic characteristics (gender, race, income, age, marital status, and education) revealed no differences among scenario conditions. Forty-four percent of the participants were female, 80% were Caucasian, 90% completed at least some college coursework, 75% of participants reported being currently employed, and 43% were single. The median age of participants was 30 years old (interquartile range 25–37), median household size was 2 persons (interquartile range 2–4), median annual income was $37,000 (interquartile range $22,000 –$62,500) and median days since last sexual encounter was 6 days (interquartile range 2–22.75).

### Procedures

The Virginia Tech Institutional Review Board approved all procedures in this study. After meeting eligibility criteria, participants read the consent statement and accepted the HIT, which was considered implied consent and documented in a database. Written informed consent was not obtained given that this process could not practicality be carried out online through Amazon Mechanical Turk. Then participants answered demographic questions before hearing one of three scenarios, which was also textually presented on the computer screen. After listening to the scenario, the Sexual Discounting Task (SDT) was completed. Participants also listened to the scenario before completing the hypothetical monetary discounting task. Questionnaires (described below) were completed last. The raw data used in this manuscript is available via the online supplementary materials.

#### Scenarios

Participants were assigned to groups and presented one of three textual and auditory scenarios prior to presentation of the SDT instructions and prior to the hypothetical monetary discounting task instructions. The researchers created these scenarios such that the number of characters and content were identical apart from than the manipulated variables. To ensure the scenario described a person with whom the participant would relate the answer to a question about the gender of their best friend was incorporated into the scenario. Also included were the participant’s age and a statement suggesting that the listener engages in similar sexual behavior to their friend.

#### Negative scenario with regret expression

“Taylor, your best friend who is also male/female and X years old and engages in sexual behavior similar to yours, just called to tell you about a social gathering s/he attended where s/he met someone s/he was interested in. They ended up having sex without using protection and Taylor expressed extreme regret. S/he said, “I knew I should have used protection that night. What was I thinking?!” Soon after the experience, Taylor experienced a sore throat, fever, rash, fatigue, headache, and muscle pain and described it as “the worst flu ever”. Taylor went to the doctor for these symptoms and tested positive for the HIV virus that causes AIDS. Taylor is profoundly devastated, afraid his/her whole life is over, and wishes s/he never made the mistake.”

#### Negative scenario

“Taylor, your best friend who is also male/female and X years old and engages in sexual behavior similar to yours just called to tell you about a social gathering s/he attended where s/he met someone s/he was interested in. They ended up having sex without using protection and Taylor expressed extreme excitement. S/he said, “I had a great time and my partner was very attractive. I’m excited to see them again!” Soon after the experience, Taylor experienced a sore throat, fever, rash, fatigue, headache, and muscle pain and described it as “the worst flu ever”. Taylor went to the doctor for these symptoms and tested positive for the HIV virus that causes AIDS. Taylor is profoundly devastated, afraid his/her whole life is over, and crying uncontrollably.”

#### Positive scenario

“Taylor, your best friend who is also male/female and X years old and engages in sexual behavior similar to yours, just called to tell you about a social gathering s/he attended where s/he met someone s/he was interested in. They ended up having sex without using protection and Taylor expressed extreme excitement. S/he said, “I had such a good time and my partner was very attractive. I can’t wait to see them again!” Soon after the experience, Taylor experienced a sore throat, fever, rash, fatigue, headache, and muscle pain and described it as “the worst flu ever”. Taylor went to the doctor for these symptoms and tested negative for the HIV virus that causes AIDS. Taylor is extremely happy and called you jumping for joy.”

#### Sexual Discounting Task (SDT)

The Sexual Discounting Task is a computerized measure of risky sexual behavior modeled from the monetary delay discounting procedure [2–4]. In this task, the participant was first presented with 60 images of individuals, both male and female of diverse age and minority statuses and then asked to identify those with whom the participant would have casual sex. A minimum of two images had to be selected. The participant was asked to identify, of the images selected, an image for each of the following conditions: 1) the person they would most like to have sex with, 2) the person they would least like to have sex with, 3) the person most likely to have a sexually transmitted infection (STI), and 4) the person least likely to have a STI. The same image could not be picked for both the most and least partner questions within a condition (i.e., STI or attractiveness). Participants were then instructed to imagine there is no chance of pregnancy and that they are single and would not be cheating on anyone if they say they would have sex with a person in one of the images (see Johnson & Bruner, 2012 for the original version of this task).

Images from each of the four conditions were then presented in a randomized order across participants. Below the image, a visual analog scale from 0 to 100 was presented, anchored by the options to have sex now without a condom (0) and to have sex at some delay with a condom (100). The participant was instructed to click the place on the visual analog scale that best represented their choice. The first question of each condition asked the participant to choose between having sex now with a condom and having sex now without a condom. The next seven questions asked the participant to make a choice between sex now without a condom and sex at some delay (1 hour, 3 hours, 6 hours, 1 day, 1 week, 1 month, 3 months) with a condom. During the first partner condition participants experienced, audio instructions were provided to ensure understanding of the task, but the audio portion of the instructions was not presented during the remaining three partner conditions.

#### Monetary Delay Discounting Task (DDT)

The DDT is a measure of future valuation that offers participants a choice between receiving a smaller, immediate amount of money or a larger, delayed amount. Delays (1 day, 1 week, 1 month, 3 months, 1 year, 5 years) were presented in a random order across participants. To detect the point of subjective equality (i.e., the indifference point), participants made choices between the $1000 larger, delayed amount and a titrated smaller, immediate amount, which were presented randomly on the left and right sides of the screen.

#### Zimbardo Time Perspective Inventory Future Orientation subscale (ZTPI)

This 13-item subscale of the Zimbardo Time Perspective Inventory measures orientation to future events. Data from one question, “I keep working at difficult uninteresting work if it will help me get ahead.” was accidentally not recorded. As a result, the total average score is calculated minus that question. High scores on the ZTPI are associated with greater orientation to the future [40].

#### HIV Risk Taking Behavior Scale (HRBS)

After completing the SDT and DDT, participants completed the 11-item HIV Risk Taking Behavior Scale. Questions about injection drug use and sexual behavior were answered on Likert scales ranging from 0–5 with different anchors for each question [41]. High scores on the HRBS indicate a greater risk of contracting HIV.

#### Short Susceptibility Scale (SSS)

The 21-item SSS is comprised of questions from the five subscales of the Multidimensional Iowa Susceptibility Scale. Questions are answered on a Likert scale from not at all or very slightly (1) to a lot (5) and the total score of all items is representative of suggestibility [42].

#### Consideration of Future Consequences Scale (CFC)

This 12–item scale measures the extent of consideration of the future consequences of current behavior, a stable trait, on a 5-point Likert scale. High scores represent more consideration of future consequences during current behavior [43].

### Data Analysis

**Fig 1:**
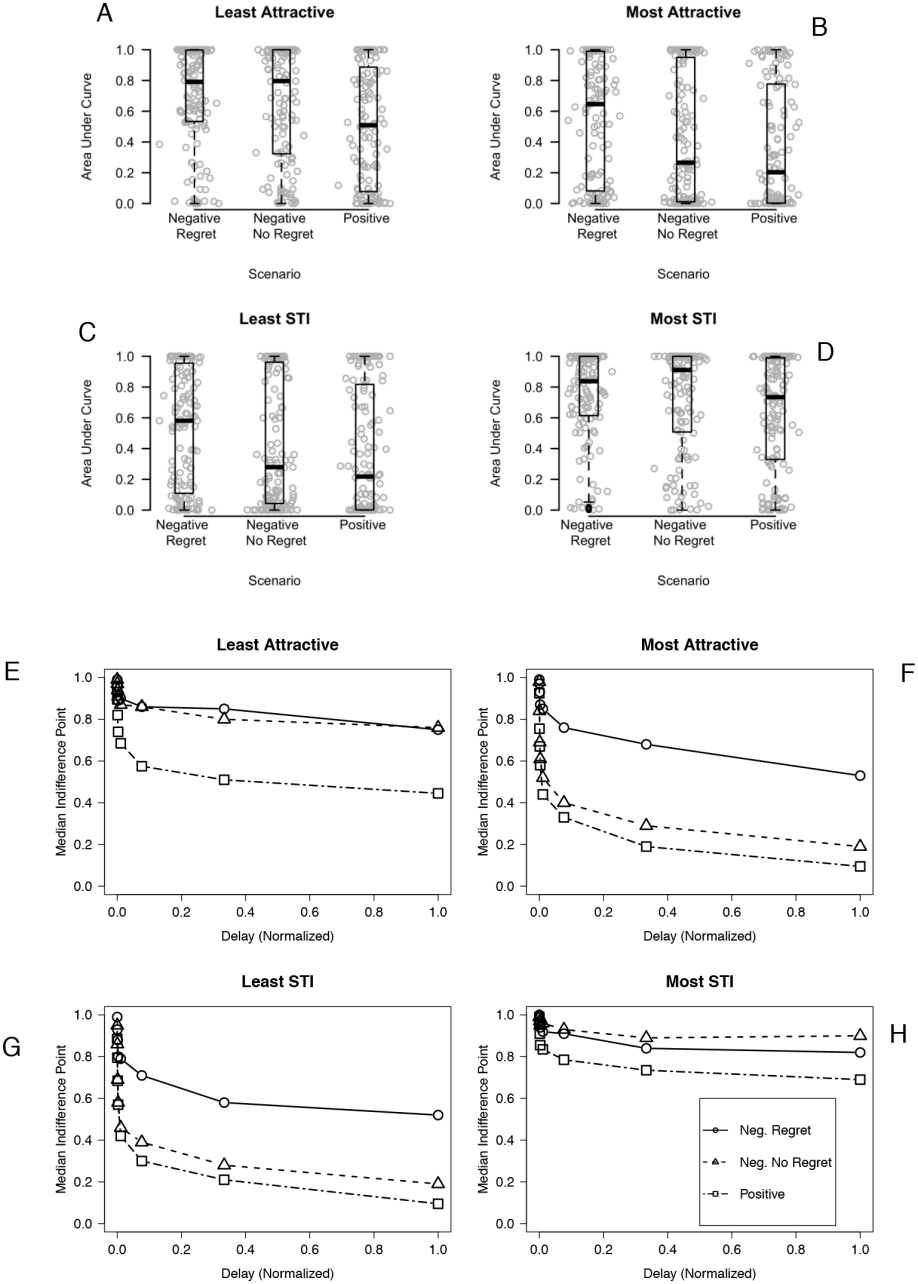
AUC values for each partner condition by scenario depicted two ways. In panels A—D, dots represent individual discounting sessions, and boxplots provide the minimum, maximum, first and third quartiles, and median. Points are horizontally jittered (i.e., a small amount of random noise is added) to enhance visibility. In panels E—H, median indifference points for each delay to condom-protected sex are represented for each scenario by partner condition.

For each participant five sets of indifference points were obtained (one set for each of the four partner conditions and one set for monetary discounting) and area under the curve (AUC) was calculated for each set of indifference points [2,44]. A lower AUC score represents a greater discounting rate and a preference for smaller, immediate rewards [44]. Examination of Fig 1 reveals a point mass at each end of the AUC distribution. Since these AUC scores violate modeling assumptions (e.g., normality of residuals), nonparametric Wilcoxon Rank Sum tests were used to compare the distribution of AUC values between scenarios in both the SDT and DDT.

Spearman correlations were calculated to examine the relationships between rate of sexual and monetary discounting and the HRBS score, ZTPI Future Orientation Scale score, number of images selected in the SDT (image count), SSS score, and CFC score. Furthermore, a comparison between HRBS/AUC correlations was performed using a permutation test to determine if the relationship (i.e., correlation) between AUC and HRBS score differed depending on which scenario the participant was assigned. This approach was chosen to avoid parametric assumptions on the distribution of AUC values and HRBS scores. The permutation hypothesis test (for a review, see [45]) is a resampling technique that obtains a p-value for the null hypothesis of zero correlation. Data are randomly reassigned to the scenarios a large number of times (here, 100,000), and differences in correlations are computed based on the randomly sampled labels. The distribution of differences from these samples approximates the null distribution (i.e., the distribution if there are no population differences in the correlations). Then, the observed difference is compared to the null distribution, and the p-value is calculated as the proportion of simulated differences that exceed the observed difference. Two-sided tests were used.

## Results

### AUC Comparison among Scenarios

Across all partner conditions, independent of scenario, lower AUC was found for males compared to females (least attractive: Z = -4.96, *p* < 0.0001; most attractive: Z = -8.04, *p* < 0.0001; least STI: Z = -6.96, *p* < 0.0001; most STI: Z = -3.12, *p* = 0.0018).

#### Least attractive

Median AUC for participants who experienced the positive scenario was significantly lower than median AUC for those who read either the negative scenario (Z = -3.34, *p* = 0.0008) or the negative scenario with regret expression (Z = -4.07, *p* < 0.0001). There was no statistically significant difference between the negative scenario with regret expression and the negative only scenario (Z = -0.61, *p* = 0.5435). Panels A and E of Fig 1 depict a higher discounting rate/less AUC for the group experiencing the positive scenario. Table 1 summarizes the AUC data by scenario for all partner conditions.

**Table 1:** Wilcoxon Rank Sum Test Results for All Partner Conditions. One star denotes significance at the 0.05 level, and two stars denotes significance at the 0.001 level.

Partner Condition	Scenario	Scenario	Score Mean Difference	Std. Err. Difference	Z	P-value
Least Attractive	Negative No Regret	Negative Regret	-5.77	9.49	-0.61	0.5435
	Positive	Negative No Regret	-31.85	9.53	-3.34	0.0008**
	Positive	Negative Regret	-38.61	9.49	-4.07	<0.0001**
Most Attractive	Negative No Regret	Negative Regret	-24.29	9.53	-2.55	0.0108*
	Positive	Negative No Regret	-16.32	9.55	-1.71	0.0876
	Positive	Negative Regret	-39.11	9.51	-4.11	<0.0001**
Least STI	Negative No Regret	Negative Regret	-18.64	9.53	-1.96	0.0504
	Positive	Negative No Regret	-16.89	9.54	-1.77	0.0769
	Positive	Negative Regret	-33.68	9.51	-3.54	.0004**
Most STI	Negative No Regret	Negative Regret	0.6765	9.48	0.07	0.9431
	Positive	Negative No Regret	-22.39	9.51	-2.35	.0185*
	Positive	Negative Regret	-23.34	9.48	-2.46	.0138*
Monetary Discounting	Negative No Regret	Negative Regret	-4.56	9.54	-0.48	0.6327
	Positive	Negative No Regret	0.64	9.56	0.07	0.9462
	Positive	Negative Regret	-3.72	9.52	-0.39	0.6961

#### Most attractive

Median AUC for participants who experienced the positive scenario and the negative scenario was lower than median AUC for those who read the negative scenario with regret expression (positive: Z = -4.11, *p* < 0.0001; negative: Z = -2.55, *p* = 0.0108). There was no statistically significant difference in median AUC between those experiencing the positive scenario and those experiencing the negative no regret scenario (Z = -1.71, *p* = 0.0876). Panels B and F of Fig 1 show less discounting/more AUC for the group experiencing the negative scenario with regret expression.

#### Least STI

Individuals who experienced the positive scenario had lower median AUC than those who experienced the negative regret scenario (Z = -3.54, *p* = 0.0004). Median AUC for individuals who experienced the negative outcome without regret expression scenario did not significantly differ from the positive (Z = -1.77, *p* = 0.0769), but approached significance compared to the negative regret outcome scenario (Z = -1.96, *p* = 0.0504). Panels C and G of Fig 1 represent less discounting/more AUC for participants that experienced the negative scenario with regret expression.

#### Most STI

Individuals who experienced the positive scenario had lower median AUC than those who experienced either scenario with a negative outcome (negative: Z = -2.35, *p* = 0.0158; negative regret: Z = -2.46, *p* = 0.0138). There was no statistically significant difference between the negative scenario with regret expression and the negative no regret scenario (Z = 0.07, *p* = 0.9431). Panels D and H of Fig 1 show more discounting/less AUC in participants that experienced the positive scenario.

#### Monetary discounting

There were no statistically significant differences in AUC calculated from monetary discounting indifference points among the scenarios in any partner condition.

### Relative Strength of HRBS/Sexual Discounting AUC Correlations by Scenario

The correlation analysis revealed that scores from the HRBS are significantly correlated with AUC in each of the partner conditions. Table 2 summarizes the relative strength of the HRBS correlations with AUC in the SDT by partner condition.

**Table 2:** Comparison of strength of Spearman correlations between HRBS and AUC by partner condition. One star denotes significance at the 0.05 level and two stars denotes significance at the 0.01 level.

Partner Condition	Scenario (Spearman correlation)	Scenario (Spearman correlation)	Two-sided p-value
**Least Attractive**	Negative No Regret (-0.2616)	Negative Regret (0.0093)	0.0308*
	Negative No Regret (-0.2616)	Positive (-0.2102)	0.6829
	Positive (-0.2102)	Negative Regret (0.0093)	0.0782
**Most Attractive**	Negative No Regret (-0.1629)	Negative Regret (0.0787)	0.0567
	Negative No Regret (-0.1629)	Positive (-0.2756)	0.3767
	Positive (-0.2756)	Negative Regret (0.0787)	0.0048**
**Least STI**	Negative No Regret (-0.1659)	Negative Regret (0.1071)	0.0279*
	Negative No Regret (-0.1659)	Positive (-0.3329)	0.1825
	Positive (-0.3329)	Negative Regret (0.1071)	0.0004**
**Most STI**	Negative No Regret (-0.2838)	Negative Regret (-.0899)	0.1169
	Negative No Regret (-0.2838)	Positive (-0.1257)	0.2019
	Positive (-0.1257)	Negative Regret (-.0899)	0.7685

#### Least attractive

The permutation test revealed a significantly stronger correlation between AUC and HRBS in the negative no regret scenario than in the negative scenario with regret expression (*p* = 0.0308).

#### Most attractive

The correlation between AUC and HRBS was significantly stronger only in the positive scenario when compared to the negative scenario with regret expression (*p* = 0.0048).

#### Least STI

Using a permutation test, the correlation between AUC and HRBS was significantly stronger in the negative no regret scenario than in the negative scenario with regret expression (*p* = 0.0279). Furthermore, the correlation between AUC and HRBS is stronger in the positive scenario than in the negative regret scenario (*p* = 0.0004). There was no statistically significant difference in the strength of the AUC/HRBS correlation between the negative no regret scenario and the positive scenario (*p* = 0.1825).

#### Most STI

The correlations between HRBS and AUC do not significantly differ in strength among the scenarios for this partner condition.

### Correlations among Measures

Correlations were analyzed to determine the strength of the relationships between AUC and several other measures of interest. Table 3 summarizes the correlations between AUC in the sexual discounting task and other measures.

**Table 3:** Spearman correlations between sexual discounting AUC and other measures of risky sexual behavior and future valuation. One star indicates significance at the 0.05 level, while two stars indicates significance at the 0.01 level.

Partner Condition	Variable	Spearman ρ	p-value
Least Attractive	Monetary AUC	0.0271	0.585
	ZTPI	0.0979	0.0482*
	Image Count	-0.1317	0.0077**
	HRBS	-0.1495	0.0039**
	SSS Score	-0.1861	0.0002**
	CFC	-0.1799	0.0003**
Most Attractive	Monetary AUC	0.042	0.3971
	ZTPI	0.095	0.0551
	Image Count	-0.3729	<0.0001**
	HRBS	-0.1074	0.0387*
	SSS Score	-0.1334	0.007**
	CFC	-0.2359	<0.0001**
Least STI	Monetary AUC	0.059	0.2344
	ZTPI	0.0647	0.1923
	Image Count	-0.2695	<.00001**
	HRBS	-0.1315	0.0112*
	SSS Score	-0.1152	0.0199*
	CFC	-0.2159	<0.0001**
Most STI	Monetary AUC	0.0159	0.7491
	ZTPI	0.0794	0.1093
	Image Count	-0.0254	0.6091
	HRBS	-0.162	0.0017**
	SSS Score	-0.1183	0.0168*
	CFC	-0.1789	0.0003**

#### Least attractive

AUC is significantly positively correlated with ZTPI score (Spearman ρ = 0.0979, *p* = 0.0482) and significantly negatively correlated with HRBS (Spearman ρ = -0.1495, *p* = 0.0039), CFC (Spearman ρ = -0.1799, *p* = 0. 0003), image count (Spearman ρ = -0.1317, p = 0.0077), and SSS score (Spearman ρ = -0.1861, *p* = 0.0002). AUC for the SDT is not significantly correlated with AUC for the DDT (Spearman ρ = 0.0271, *p* = 0.585).

#### Most attractive

AUC for this condition of the SDT is significantly negatively correlated with HRBS (Spearman ρ = -0.1074, *p* = 0.0387), CFC (Spearman ρ = -0.2359, *p* < 0.0001), image count (Spearman ρ = -0.3729, *p* < 0.0001), and SSS score (Spearman ρ = -0.1334, *p* = 0.0007). AUC for the SDT is not significantly correlated with AUC for the DDT (Spearman ρ = 0.042, *p* = 0.3971), or with ZTPI score (Spearman ρ = 0.095, *p* = 0.0551).

#### Least STI

AUC for this condition of the SDT is significantly negatively correlated with HRBS (Spearman ρ = -0.1315, *p* = 0.0112), CFC (Spearman ρ = -0.2159, *p* < 0.0001), image count (Spearman ρ = -0.2695, *p* < 0.0001), and SSS score (Spearman ρ = -0.1152, *p* = 0.0199). It is not significantly correlated with AUC for the DDT (Spearman ρ = 0.0590, *p* = 0.2344) or with ZTPI score (Spearman ρ = 0.0647, *p* = 0.1923).

#### Most STI

AUC for the SDT is significantly negatively correlated with HRBS score (Spearman ρ = -0.1620, *p* = 0.0017), CFC score (Spearman ρ = -0.1789, *p* = 0.0003), and SSS score (Spearman ρ = -0.1183, *p* = 0.0168). It is not significantly correlated with AUC for the DDT (Spearman ρ = 0.0159, *p* = 0.7491), ZTPI score (Spearman ρ = 0.0794, *p* = 0.1093), or image count (Spearman ρ = -0.0254, *p* = 0.6091).

## Discussion

Consistent with the key hypothesis, this investigation found that reading a scenario about the risky sexual behavior of a similar individual that resulted in a negative outcome and with expression of regret influences hypothetical sexual discounting for condom-protected sex. In the least attractive and partner most likely to have a sexually transmitted infection conditions of the SDT, participants were more likely to choose condom-protected sex after reading the negative scenarios with and without regret expression compared to those that read the positive scenario. A difference was found for groups experiencing regret in the most attractive and partner least likely to have a sexually transmitted infection conditions, where participants were more likely to choose delayed condom-protected sex after experiencing the negative regret expression scenario. The larger differences seen with the experience of regret in these particular partner conditions are consistent with previous literature reporting larger differences between controls and opioid-dependent women in the same two partner conditions [8].

Importantly, the questionnaire measure of risky sexual behavior, the HRBS, was negatively correlated with AUC in the SDT in all partner conditions (i.e., individuals that waited longer for condom-protected sex, had lower self-reported risk of contracting HIV). Thus, the responses in the SDT are related to self-reported real world behavior, which has been previously reported [2]. Interestingly, no significant difference between the relative strength of this correlation was found in the partner condition for the image chosen as most likely to have a sexually transmitted infection suggesting that the scenario did not influence the relationship between variables, but instead the relationship was likely a result of the partner condition itself. Importantly, monetary discounting was not significantly different between any groups, which demonstrates that the scenarios have a differential effect on different discounting types. Perhaps then, this selective effect on the SDT may be more reflective of real world sexual behavior. Monetary discounting was not correlated with the HRBS or any of the supplementary measures. The correlations between the SDT responses and the supplementary measures were significant, but of small magnitude in most SDT partner conditions.

Antecedent manipulations occurring prior to hypothetical monetary discounting have been shown to both alter behavior transiently [5] and for an extended period of time. With monetary discounting, decreases have been reported for up to a month after reading a financial guide [46]. Perhaps, scenarios like those reported here could change sexual risk taking in naturalistic settings similar to a previous report [37]. In that study, participants who were asked to focus on anticipated feelings after unsafe sex often reported regret and were more likely to report using a condom up to 5 months after participation.

This experiment illustrates two important phenomena: the power of regret expression and the importance of social learning. Compared to the other scenarios, the negative regret scenario in the current study may have increased the salience of regret or the realization that unprotected sex could lead to negative emotions. Likewise, social learning theory conjectures that people are influenced by and learn from the behaviors of others in their presence [47]. This research expands that idea to suggest behavior is dependent not only the presence of another, but also upon stories about another individual. Study results suggest that hearing a story about a friend may be a more powerful experience than hearing basic information about risky sexual behavior, present in many current efforts to increase HIV risk prevention. Perhaps, exposure to cues about a similar individual or oneself could change risky sexual behavior, in and out of the laboratory and may function as an effective adjunctive therapy when combined with current strategies.

Several limitations of this study must be acknowledged. While work has been done to examine whether decisions for hypothetical, real, and potentially real monetary rewards are similar [39,48], comparisons of hypothetical and real sexual discounting have not been reported. An investigation of this sort would be difficult and perhaps unethical (i.e., providing an individual with a real sexual outcome). Also inherent in this procedure, participants were not presented with a choice not to have sex with the person in the image for each partner condition. As Johnson & Bruner (2012) point out, this option was implied in the first part of the SDT when participants choose images of people they would have sex with. In the current experiment, participants were required to choose two images to continue participation. If this constraint were not present, the lowest level of sexual risk (i.e., choosing no images) could have been assessed. Nonetheless, previous investigation using the SDT that did not enforce the same criteria, reported that all participants chose at least one image [2]. Rooted in the development and novel status of the scenarios used in the current study, demand characteristics could have modified the results.

Last, the experimental design of the current study was unbalanced such that we did not assess the effect of a positive scenario with expression of regret, thus the effects of that combination remains to be determined. Relatedly, a neutral condition was not presented to a separate group of participants for comparison, which could have functioned as a control condition given that the positive scenario may in fact increase immediate sexual gratification. However lack of a neutral or positive scenario with regret expression condition does not preclude comparison of the three conditions present in the current study and the differential effects produced by the positive and negative consequences and the selective effect of the expression of regret on responses in specific partner conditions is apparent. Development and inclusion of an appropriate neutral conclusion in future studies could bolster interpretation of the effect.

In conclusion, the SDT as a relevant laboratory measure and the framing of information to include the expression of regret may be relevant for prevention of risky sexual behavior. Perhaps scenarios including regret expression would be more likely than education efforts to prevent sexual risk, however this would entail considerable more study. For example, assessment would be necessary to determine if the effect translates to behavior change in naturalistic conditions and determination of the effect duration is necessary. Moreover, different biases could be added to the scenarios to discern if exposed individuals would engender less risky sexual behavior. In any case, the SDT and the use of framing with scenarios opens up a potentially useful approach to the study of sexual risk and provides a procedure to examine interventions in a limited way prior to clinical trials.

## Supporting Information

S1 Data Set Raw data set used for analyses in this manuscript. (CSV)

S1 Data Dictionary Definitions for data labels. (XLSX)
